# Exploring robot-led activities between people living with dementia and family care partners

**DOI:** 10.3389/frobt.2026.1772079

**Published:** 2026-04-28

**Authors:** Jirachaya Fern Limprayoon, Debasmita Ghose, Kayla Matheus, Paula V. Enriquez, Michal A. Lewkowicz, Moon Hwan Kim, Austin Narcomey, Natnaree Proud Ua-Arak, Andy Cheng, Chayan Sarkar, Joan K. Monin, Brian Scassellati

**Affiliations:** 1 Social Robotics Lab, Computer Science Department, Yale University, New Haven, CT, United States; 2 Robotics and Autonomous Systems, TCS Research, Delhi, India; 3 Social Gerontology and Health Lab, Yale School of Public Health, Yale University, New Haven, CT, United States

**Keywords:** care partner support, dementia, joint activities, social influence, socially assistive robots

## Abstract

**Introduction:**

While shared activities foster connection between people living with dementia (PLWD) and their care partners, emotional distress and daily caregiving responsibilities often make them difficult to initiate. This paper investigates the adaptation of a socially assistive robot, Ommie, to guide shared deep breathing and singing activities for these pairs.

**Methods:**

We refined the robot’s behaviors through two interaction design sessions with people living with dementia and care partners, mediated by an occupational therapist. In a subsequent study with 17 pairs, participants engaged in deep breathing and singing activities with the robot as well as in-session semi-structured interviews, and we conducted post-hoc video analysis to explore their interactional dynamics.

**Results:**

Participants reported the interactions as easy to follow, calming, and familiar. Post-hoc video analysis revealed patterns of intimacy and synchrony, including frequent physical touch, mutual gaze, and rhythmic coordination. We also observed instances of personal memory recall and a playful atmosphere, in which pairs often used humor as a coping mechanism after deviations from the robot’s instructions.

**Discussion:**

From our observations, we discuss three design opportunity spaces: the robot as the focus for synchronization, as an instrument of joint play, and as a source of familiarity versus variety.

## 1 Introduction

Individuals living with dementia routinely encounter challenges that include anxiety, depression, and fluctuations in mood (Rippon et al., 2020). These challenges often stem from increasing difficulties in communication, leading to isolation and emotional distress as the condition progresses. At a certain point, individuals living with dementia will progress to a stage requiring a regular care partner such as a spouse, child, or nurse. These care partners also experience significant emotional stress due to heavy caregiving responsibilities and limited external support (Chunga et al., 2021; Ward-Griffin et al., 2012; Scher et al., 2023). Participating in wellness activities together, such as music therapy and mindfulness exercises, has been shown to uplift mood, alleviate anxiety, and strengthen emotional bonds between individuals with dementia and their care partners (Baird and Samson, 2015; Chu et al., 2014; Russell-Williams et al., 2018; Phinney et al., 2007). However, initiating and structuring such interactions can be challenging for care partners due to the breadth of daily responsibilities and the acute time constraints inherently associated with caregiving (Chiao et al., 2015).

One promising option for supporting interactions between people living with dementia and care partners is the use of social robots. Multiple social robots have already successfully guided wellness and social activities in adjacent health contexts and populations (Matheus et al., 2025b; Thomas et al., 2025; Adamson et al., 2021). A robot that mediates wellness activities for people living with dementia and care partners to participate together as a social activity could further enhance positive support for both individuals. Social robotics research has also explored the use of robots to enrich the wellbeing of older adults [e.g. (Khosla et al., 2013; Randall et al., 2023; Li et al., 2023)] and support people living with dementia in clinical and home settings. [e.g. (Ghafurian et al., 2021; Moyle et al., 2017; Darragh et al., 2017)].

While socially assistive robots have shown promise in dementia care, prior research–even when involving care partners–has largely focused on assisting the person living with dementia, treating any benefits to the care partner as secondary rather than actively supporting them or their mutual connection [e.g. (Liang et al., 2017; Wang et al., 2017)]. Shifting this focus, our work seeks to explore how a social robot outside controlled lab settings might guide wellness activities for persons living with dementia (PLWDs) and their care partners (CPs) to participate together, drawing on Bennett et al.’s interdependence framework to approach dementia as a shared, collaborative experience (Bennett et al., 2018). While prior work has explored conceptual guidelines for how robots can act to support people living with dementia and care partners (Lee H. R. et al., 2023), we are among the first to deploy a social robot in this connective role. We first worked collaboratively and iteratively with experts and members of a local care facility to adapt the design of the Ommie robot (Matheus et al., 2025b) – initially designed for young adults to practice deep breathing–to the needs of people living with dementia and care partners (Section 3.2). We identified a strong desire for a sense of togetherness from PWLDs and their CPs. Practically, the robot required additional verbal and visual modalities of instruction, tactile improvements, and increased positive reinforcement for this population. Additionally, we identified and developed tailored robot interactions for two wellness activities: deep breathing and singing (Section 3.3).

**FIGURE 1:**
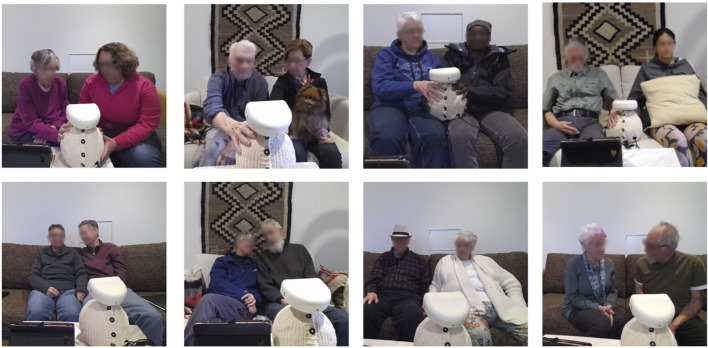
Observational Study Highlights: People living with dementia and their care partners engaging with Ommie during breathing (top) and singing (bottom) activities.

We evaluated the potential of our participatory design interaction set through a qualitative observational study with 17 pairs of people living with dementia and care partners (Figure 1). The wellness activities (Section 3.4) were conducted at the host facility, which served as either a residence or a site for community classes. All sessions were audio- and video-recorded to capture the interactions. Our exploratory measures include semi-structured interviews and *post hoc* video coding of participant behaviors in response to robot actions (Section 3.7). The interviews captured participants’ perceptions of the robot, their emotional responses, and their feedback on potential home integration.

Our results show that PLWDs and their CPs were both able to successfully engage with the robot and with each other. Participants describe the robot-led activities as calming, familiar, and naturally prompting recollection of past memories. We additionally observed promising signs of social connection during robot use, evidenced by synchronous gaze, touch, movement, and humor or play. Participants also suggested several improvements to the robot experience, focusing on interactivity and personalization. Based on our findings, we propose and discuss three design opportunities for HRI researchers seeking to support people living with dementia and care partners (Section 5): (a) the robot as a focus for synchronization; (b) the robot as an instrument of joint play; and (c) the robot as a source of tension between familiarity and variety. Overall, our findings serve as a starting point for continued exploration of how social robots can support shared engagement in care relationships between people living with dementia and their care partners.

## 2 Background and related work

### 2.1 Supportive measures to care for adults living with dementia

Care for people living with dementia spans a wide range of interventions, from professional clinical to home-based support, depending on each person’s needs. People living with dementia often engage in professional-led interventions such as cognitive stimulation therapy (Orfanos et al., 2021; Spector et al., 2011). For individuals with advanced care needs, residential facilities provide essential 24/7 clinical support that is difficult to replicate at home (Zimmerman et al., 2005). However, there is a growing emphasis on home-based care that allows people living with dementia to remain in familiar surroundings when full-time professional assistance is not yet required. Home-based interventions include guided wellness activities, such as mindfulness (Russell-Williams et al., 2018; Shim et al., 2021), music (Baird and Samson, 2015), light exercise (Jensen et al., 2015; Fasola and Matarić, 2013), and storytelling (Basting, 2020), which all serve as vital tools for improving mood and strengthening social connections.

Building on prior research using social robots for wellness, our study explores how a robot can lead sessions in which both the person living with dementia and their care partner engage as active participants.

### 2.2 Robots for people living with dementia

Researchers have increasingly explored the use of social robots to support people living with dementia (PLWD) across a wide range of clinical and home settings (Ghafurian et al., 2021). Much of the existing work explores robots designed to alleviate neuropsychiatric symptoms (Saragih et al., 2021), monitor cognitive performance (Cruz-Sandoval et al., 2020; Luperto et al., 2024), or provide direct functional assistance for daily routines (Akinrintoyo and Salomons, 2025; Begum et al., 2013). For instance, functional robots help individuals manage memory-intensive tasks, such as locating misplaced objects (Shah et al., 2023; Tuyen et al., 2026) or providing reminders for scheduled activities (Wang et al., 2017). Beyond these practical aids, social robots have been used to facilitate in-person communication for the individual by offering real-time memory support during social interactions (Zhou et al., 2021) or acting as mediators that help older adults record and share sensitive feelings with their families (Noguchi and Okada, 2023).

Recent advancements in conversational AI and telepresence have further expanded the role of robots as social companions, though these often focus on connecting individuals across separate locations. Telepresence robots show promise in helping PLWD maintain social connections with distant family members, acting as a remote bridge when physical presence is not possible (Moyle et al., 2017). Similarly, the integration of large language models has enabled robots to act as conversational companions to mitigate loneliness, typically through one-on-one engagement with individuals (Irfan et al., 2024; Bassett et al., 2026). While valuable, these interventions generally position the robot as a substitute for human presence or as a tool for remote communication (Moyle et al., 2017; Irfan et al., 2024), posing a risk of reducing meaningful in-person interaction between people living with dementia and care partners.

Building upon prior work demonstrating the feasibility of using social robots for people living with dementia, we utilize a social robot to initiate and scaffold wellness activities (in this case, deep breathing and singing) for people living with dementia and their care partners.

### 2.3 Robots addressing needs of the care partners of the people living with dementia

Rather than focusing on the person living with dementia in isolation, care partners should be included in the design process to ensure robots support both individuals, recognizing that these partners are pivotal to the care dynamic and possess needs distinct from those living with dementia (Chunga et al., 2021; Lee H. R. et al., 2023). People living with dementia and care partners often perceive robots differently: for example, a person living with dementia may value a companion robot primarily for the intuitive joy and emotional comfort it provides, whereas a care partner evaluates it based on its practical durability, ease of use, and how it fits into their care routine (Yamato et al., 2023). To address these differences, some researchers have used end-user programming to empower clinical caregivers to create personalized robot behaviors tailored to their patients’ specific cognitive needs (Kubota et al., 2020; Kooij-Meijer et al., 2026). Recent participatory design studies have also explored how social robots can assist older adults while respecting the distinct desires of their caregivers (Dosso et al., 2023; Randall et al., 2023; Lee H. R. et al., 2023). These studies suggest that robots can strengthen the connection within the pair, provided they address the caregiver’s need for support without compromising the autonomy of the person living with dementia.

Inspired by this need to support both parties, we conducted an iterative design process involving people living with dementia and care partners as active stakeholders. We facilitated two interaction design sessions to gather direct feedback on the adaptation of the social robot Ommie (Matheus et al., 2025b). By including both members of the pair in these early design stages, we refined the robot’s behaviors to benefit both parties simultaneously. These interaction sessions allowed us to move beyond individual-centered design and prepare the system for the shared, in-person interactions explored in our user study.

### 2.4 Robot-mediated social interaction between mixed-ability teams

In recent years, research has shifted from robots serving individual users to robots actively facilitating richer human-human interactions (Sebo et al., 2020). This “interaction-shaping” role allows robots to strategically influence social patterns to promote connection, mediate conflict, and enhance group dynamics (Gillet et al., 2024). This form of facilitation is particularly vital for enabling equity and agency in mixed-ability teams where participants may have differing cognitive, sensory, or social capacities. For example, robots have been shown to improve communication between children with ASD and their care partners (Scassellati et al., 2018) and have successfully balanced decision-making input in groups with mixed visual abilities (Neto et al., 2023).

In the context of dementia, robots have been shown to increase verbal engagement among groups of people living with dementia (Thompson et al., 2017; Preston et al., 2024). However, when considering the partnership between care partners and people living with dementia, technology has mostly focused on support for functional tasks, such as handwashing or schedule management (Begum et al., 2013; Preston et al., 2024; Liang et al., 2017; Casey et al., 2020). While previous community-based participatory design studies involving both people living with dementia and their informal care partners have highlighted the potential for social robots to foster meaningful shared moments within the pair (Moharana et al., 2019; Lee H. R. et al., 2023), these concepts have yet to be tested through in-person sessions with a physical robot.

Building on these principles, we applied the social robot Ommie (Matheus et al., 2025b) to guide activities that a person living with dementia and their care partner engage in together. Our approach treats both parties as active participants: the robot guides breathing and singing exercises designed specifically as a shared experience between the person living with dementia and their care partner.While iterative designs for such social robots have been proposed, to our knowledge, this work is among the first field applications of a social robot in this role. To explore the interaction dynamics that emerge in these shared environments, we followed our interaction design sessions with a user study involving 17 pairs.

## 3 Methodology

To investigate the impact of robot-facilitated shared activities, we first conducted design sessions with people living with dementia (PLWD) and their care partners. These sessions informed the development of tailored Deep Breathing and Singing activities, which were subsequently evaluated with 17 participant pairs. We collected data through semi-structured interviews to capture immediate perceptions and performed *post hoc* qualitative coding of both interviews and video recordings to analyze interaction dynamics.

### 3.1 Robotic system

Ommie is a socially assistive robot custom-built from a mix of 3D-printed and off-the-shelf parts to guide deep breathing practices (Matheus et al., 2025b). Ommie provides haptic feedback through capacitive touch sensors and the mechanical expansion and contraction of its body. This motion follows a deep breathing pattern, with each cycle including an inhale, a hold, an exhale, and a final hold, with customizable durations for each phase. When a user places their hands on the robot, they can physically feel the robot’s breathing patterns and synchronize their breathing accordingly (Matheus et al., 2025b). Ommie has a head that can nod, digital eyes that can open and close, and an internal speaker to facilitate deep breathing interactions. Ommie is outfitted with a microfiber sweater.

We selected Ommie for this work due to the robot’s prior positive performance in reducing stress in young adults experiencing anxiety (Matheus et al., 2022) and in children undergoing risky medical procedures (Chun et al., 2025). These applications establish a precedent for successfully deploying Ommie with vulnerable populations and supporting a wellness activity studied with both PLWDs (Lee S.-H. et al., 2023) and their CPs (Tahsin et al., 2021). Additionally, we posited that the robot’s non-verbal, haptic modality would make it uniquely suitable for PLWD, as it allows users to follow along with deep breathing without having to remember the verbal instructions given at the beginning. Furthermore, touch interactions are typically preferred by older adults, who are less familiar with technology compared to alternatives such as graphical user interfaces (GUIs) (Polak et al., 2018; Rossato et al., 2021).

### 3.2 Interaction design

To gain a deeper understanding of the needs of people living with dementia, we partnered with a non-profit organization specializing in dementia care that offered different levels of care and services to individuals living with dementia and their care partners. The facility is privately owned and provides residential facilities for over 120 people living with different stages of dementia. The facility also offers residential neighborhoods that range from assisted living, where people living with dementia can reside with their care partners, to skilled nursing, where a medical team regularly supervises their health. The facility also offers classes for adults interested in maintaining their brain health, for facility residents, and for other older adults in the community. We collaborated with the board members of their partnership network (comprised of the Chief Strategy Officer, an occupational therapist, people living with dementia, and family care partners) across two interaction design sessions.

#### 3.2.1 Design session I

**FIGURE 2:**
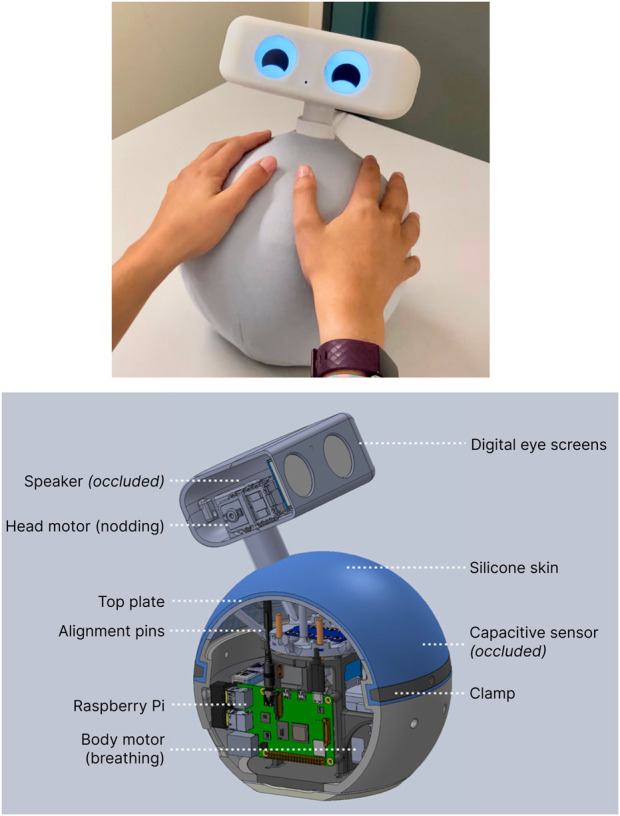
Ommie Design Overview. (Left) The physical embodiment of Ommie. (Right) A CAD cross-section illustrating internal hardware components. Reproduced from Matheus et al. (2025b).

Our first session focused on seeing how well the original Ommie system, shown in Figure 2, worked for people living with dementia exactly as it was, without any changes. The robot guided deep breathing by expanding and contracting its body and providing rhythmic chime cues. Before the session, the occupational therapist informed the researchers that people at the care facility were most familiar with a 4-4-4-4 ‘box breathing’ pattern (4-s intervals for inhaling, holding, exhaling, and holding); consequently, the robot was pre-programmed to follow this cycle.

The interaction was designed as a one-on-one experience for the person living with dementia, with the care partner simply observing. The 1-h feedback session took place in a conference room, mediated by an occupational therapist. After a researcher introduced the device, participants were invited to hold the robot for three breathing cycles. Once the participant indicated they were ready to begin, a researcher initiated the scripted cycle via a laptop. Beyond pressing “start” to trigger the pre-programmed sequence, the researcher had no further control over the robot’s behavior and did not intervene during the session. Four people living with dementia (2 female, 2 male) engaged with the robot, while three care partners (3 female) observed. Actionable feedback can be summarized as follows:

F1 Care partners emphasized the importance of shared engagement, noting they want to be active participants together and maintain their relationship with the person living with dementia, rather than have the robot replace that connection.
F2 The microfiber sweater lacked a soothing tactile sensation, making it less conducive to relaxation.
F3 Clear verbal instructions during breathing would be essential for people living with dementia.
F4 Both people living with dementia and care partners suggested that some kind of visual feedback would be helpful.

#### 3.2.2 Design session II

Based on insights from Design Session I, several hardware refinements were implemented to better suit the users’ needs. To enhance tactile appeal, the robot’s exterior was updated from microfiber to a knitted sweater [F2]. Additionally, a 10-inch tablet was integrated to serve as a centralized control interface and visual aid. This allowed users to wake the robot, which would transition from closed to open eyes when they pressed a button on the tablet, selecting their preferred activities, and following visual guidance throughout the session [F4]. To provide clearer guidance, Ommie now begins the session with a verbal self-introduction [F3].

To transition from an individual to a shared experience, we modified the deep breathing interaction to engage both the person living with dementia and their care partner [F1]. The robot initiated this joint participation by saying, “Can both of you put your hands on my sweater? That way you can feel me breathe and follow along,” encouraging both the person living with dementia and the care partner to follow its rhythmic movements together. During the activity, the robot performed a 4-4-4-4 box breathing pattern. To improve the introduction to deep breathing activity [F3], the robot supplemented its physical expansions and auditory chimes with spoken prompts (“inhale,” “hold,” “exhale,” “hold”) during the first cycle.

Alongside updates to the robot’s body texture and deep breathing activity, we introduced a new singing activity based on established dementia care practices (Morris et al., 2024; Moharana et al., 2019). Similar to the breathing activity, Ommie introduced itself at the start of the activity. It then sang the first portion of *Sweet Caroline* while nodding, blinking, and displaying lyrics on the tablet. When the song got to the chorus, Ommie stopped singing and encouraged the couple to sing together by saying “Now both of you sing.” When the song finished, Ommie then said, “Let’s sing together from the top with all of us.” Then the song restarted from the top, and Ommie started singing again from the beginning to the end of the song. Both the singing and breathing activities concluded with Ommie providing positive reinforcement by saying, “Great job!” to signal the end of the activity [F3]. Notably, the robot exhibited the same behaviors toward both participants, without distinguishing between the person living with dementia and the care partner, to encourage equal participation.

To explore how well the updated system would meet the needs of people living with dementia and their care partners, we conducted a 2-h session using appreciative inquiry (Cram, 2010). This inclusive framework has been shown to foster creativity through positive questioning and to build on participants’ existing strengths to establish a shared vision. This session involved a romantic couple (a person living with dementia and their care partner) from the Design Session I who volunteered to provide detailed feedback, mediated by the same occupational therapist from the previous session. To begin the session, the researcher set the robot to a “sleep” state before participants walked in the room. From this point, the interaction was fully scripted and autonomous. Participants initiated the interaction by pressing a button on the tablet to “wake” the robot, causing its eyes to transition from closed to open. Participants were then instructed to choose “deep breathing” option first by pressing the button on the tablet screen. During this session, the robot encouraged the couple to perform three cycles of deep breathing together as they placed one hand on each of its shoulders. When the deep breathing activity concluded, the participants were interviewed about their experience of deep breathing activity. After that, participants were instructed to press “singing” option on the tablet screen, which started the “Sweet Caroline” singing activity.

After interacting with the updated version of the system, the care partner appreciated that the tablet application was very simple and minimal. They also appreciated how the system was tailored to older adults, including loud volume, large font sizes, high-contrast buttons, and clear verbal instructions throughout the interaction. They also noted that during the singing activity, the tablet displayed a single line of lyrics to avoid overwhelming the person living with dementia. The occupational therapist noted that a home-like setting, rather than a formal boardroom, is essential for eliciting natural behaviors and observing more authentic interactions. Alongside their positive feedback, the couple also offered the following feedback that needed to be addressed prior to the user study:

F5 The couple recommended starting with a standalone demonstration by the robot, followed by an extension of the deep breathing exercise to four or five total cycles. They specifically suggested that verbal instructions should continue through the first few rounds, rather than ending after the first.
F6 The couple suggested giving participants the option to repeat the same songs during the study, noting that a strong personal or emotional attachment to specific music often enhances the experience.
F7 While the couple liked that the robot nodded along, the care partner felt the movement was too frequent, making the robot seem too social. They were concerned this might distract the person living with dementia and pull their attention away from the care partner.

### 3.3 Activities

Drawing on the feedback from Design Session II (Section 3.2.2), we refined both core activities to better support the pairs of people living with dementia and care partners. Specifically, we extended the verbal guidance and auditory chimes in the breathing exercise through the first three rounds to help participants establish the pattern [F5]. We adjusted the robot’s nodding to occur intermittently, using regular pauses to prevent the robot from overshadowing the care partner and drawing too much of the person living with dementia’s focus [F7]. We also added four more song options. The finalized activities for the study are described below:

#### 3.3.1 Deep breathing

**FIGURE 3:**
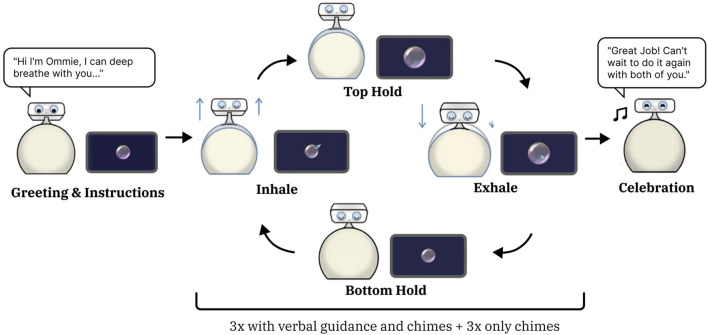
The Deep Breathing Activity. A diagram of the Ommie robot performing the deep breathing activity in our study. The robot would greet users, provide instructions, and cycle through six deep breathings, half with verbal instructions. A side tablet visualized a circle expanding and contracting at the same time as the robot’s physical body expansion. The robot concluded the activity with a short celebration.

When the deep breathing activity was selected, the robot prompted participants to place their hands on its sweater. Following a 3-s countdown, Ommie guided the pair through a 4-4-4-4 box breathing pattern. To provide visual aid, the tablet displayed a synchronized visual of a circle expanding and contracting. While the full exercise consisted of six breathing cycles, the robot provided the verbal guidance and auditory chimes through the first three rounds to establish the breathing pattern. For the remaining three cycles, the robot provided chimes only, allowing for a more meditative experience. The interaction concluded with Ommie performing a celebratory motion and saying, “Great job!”, as illustrated in Figure 3.

#### 3.3.2 Singing

**FIGURE 4:**
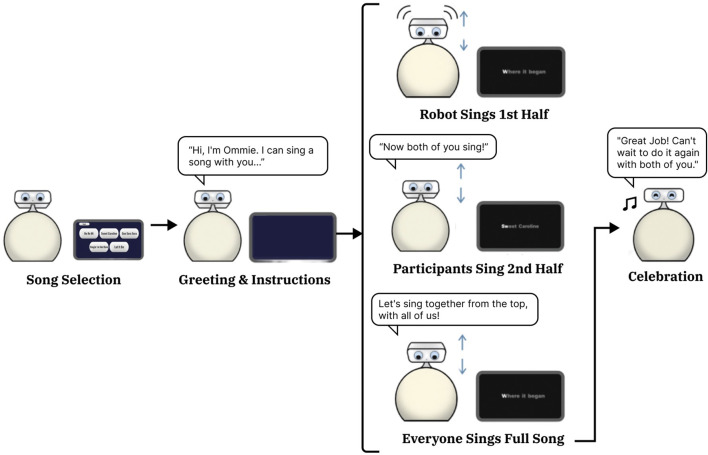
The Singing Activity. A diagram of the Ommie robot performing the singing activity in our study. After a user selected a song, the robot would greet users, provide instructions, and sing half the song. The robot would prompt the PLWD and CP to sing the rest of the song themselves together. After the song completed, the robot would prompt that all three agents (two humans and one robot) sing the song again together. The robot concluded the activity with a short celebration.

When the singing activity was selected, the tablet presented five song options for the pair to choose from: *Do Re Mi, Singing in the Rain, Let it Be, Que Sera Sera,* and *Sweet Caroline*. These tracks were curated in consultation with the facility’s music therapist to ensure familiarity for the target age group. After a selection was made, the robot sang the song from the beginning while blinking and nodding. The robot nodded intermittently, pausing at regular intervals throughout the song. When it got to the chorus, the robot then played instrumental background music and encouraged the participants to sing from the chorus to the end of the song while it continued to display lyrics on the tablet. Once the song restarted, the robot invited both the care partner and the person living with dementia to sing the entire piece together. Following feedback on the importance of musical attachment, participants were asked to repeat this activity three times. They were given the option to repeat the same song or select a different one [F6]. The interaction ended with a celebratory motion and a verbal “Great Job!”, a sequence illustrated in Figure 4.

### 3.4 Study protocol

**FIGURE 5:**
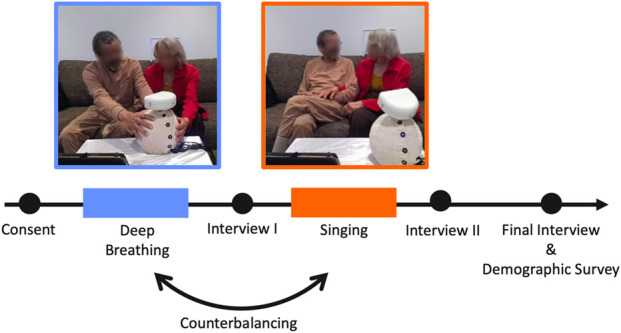
Study Protocol. Participants first verbally consent to participate in the study before engaging in either a Deep Breathing or Singing interaction. At the end of this first interaction, they participate in a semi-structured interview. Next, they engage in the alternate interaction (Singing or Deep Breathing), not repeating their first activity, and subsequently complete another semi-structured interview. Both of these post-activity interviews use the exact same structure and set of questions. At the end of the study, participants answer questions about the overall experience in an end-of-study interview and fill out a demographics survey.

The study was conducted at the same care facility, detailed in 3.2, where pairs (a person living with dementia and their familial care partner) engaged in a structured 1-h session to gather their immediate reactions to robot-facilitated activities. Following previous feedback, we conducted the study in a wellness room designed to mimic a home living area, complete with a couch and coffee table. Occupational therapists identified this familiar atmosphere as crucial for eliciting authentic, comfortable interactions. The study protocol is organized as shown in Figure 5.

**FIGURE 6:**
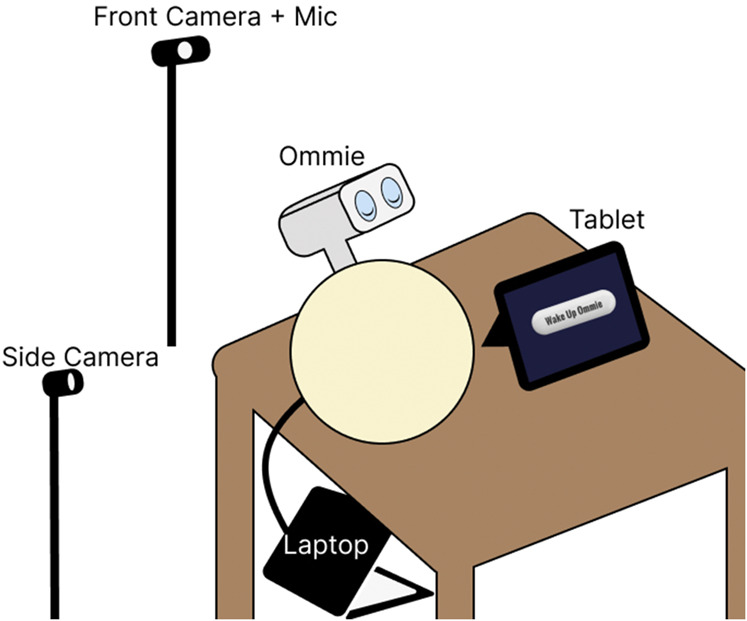
Hardware Setup. Social robot Ommie is placed on a table adjacent to a tablet. A camera with a microphone pointed at the participants is placed behind the robot setup to record the interaction. A laptop controlling the interaction is placed on the floor. Another camera is placed to the side to capture a different viewpoint.

Upon arrival in the common area, each pair of participants (a person living with dementia and their care partner) was asked to provide verbal consent. After agreeing to participate, they were directed to the wellness room where our system was set up, allowing both to sit facing the robot, which was in a pre-setup sleep state with its eyes closed, and a tablet on a table. The tablet served as the starting point for the interaction, featuring a single button labeled ‘Wake Up Ommie.’ All sessions were audio- and video-recorded using front- and side-facing cameras, as shown in Figure 6.

The researcher provided initial instructions, after which participants initiated the pre-programmed activities by pressing the corresponding buttons on the tablet. They were first instructed to wake the robot up by touching the button on the tablet screen. They were subsequently assigned to perform one of two activities, deep breathing or singing, with the order counterbalanced across sessions to control for potential order effects. Immediately following the first activity, participants completed a semi-structured interview about their impressions of what they had just done: deep breathing or singing. The researcher then instructed them to begin the second activity by pressing the corresponding button on the tablet for the remaining activity. Upon completion, a second semi-structured interview was conducted to capture their immediate impressions of that activity. To conclude the session, we conducted a semi-structured interview to explore the potential for integrating the intervention into their daily living environments and administered a demographic survey to gather participant information. Each participant received $20 as compensation.

### 3.5 Participants

We recruited 17 pairs of older adults in which at least one individual exhibited signs of cognitive change consistent with dementia, regardless of formal diagnostic status. Recruitment was a collaborative effort with the care facility’s community engagement and education teams, utilizing wellness class advertisements, door-to-door outreach, word of mouth, and direct invitations. Notably, this group included three pairs from our previous design phases: P4 (Sessions I and II), as well as P3 and P7 (Session I only). Of the 17 pairs, 15 consisted of an individual living with dementia and their care partner; in the remaining 2 pairs, both individuals were living with dementia, with one still serving as the other’s care partner. The people living with dementia in these pairs either resided in the assisted living neighborhoods of the facility or commuted frequently to the facility for classes and social activities during the day. We did not include any participants who resided in the skilled-nursing neighborhoods due to risk-management considerations. Regarding relationship types, 14 pairs were spouses or partners (12 male-female and 2 female-female), and 3 pairs were inter-generational (2 mother-child and 1 father-child). The average age of care partners was 72 years (minimum: 44, maximum: 90, SD: 10.6). Similarly, the average age of persons living with dementia was 78 years (minimum: 64, maximum: 93, SD: 7.2).

We also conducted an ADL/IADL assessment for the people living with dementia in each pair. This assessment, developed by the Dementia Care Aware program at the University of California, San Francisco (Dementia Care Aware, 2023), measures our participants’ functional abilities. We use an adapted version of the original ADL/IADL checklist, the Katz Index of Independence in ADL (Katz et al., 1963), and the Lawton and Brody IADL Scale (Lawton and Brody, 1969). The checklists were designed to assess a person’s ability across basic self-care and higher-level independent living activities. For each item, we asked whether the person could perform the activity independently (scored as 1) or needed help (scored as 0). Adults living with dementia in the study had an average Activity of Daily Living (ADL) score of 5.12 (minimum: 1, maximum: 6, SD: 1.41) and an Instrumental Activities of Daily Living (IADL) score of 1.76 (minimum: 0, maximum: 6, SD: 1.89).

### 3.6 Measures

We conducted semi-structured interviews using the questions listed in Table 2 immediately after each activity and again at the end of the session. These interviews captured immediate impressions of the robot activities and the system’s potential for in-home use. Specifically, participants were asked whether they would be willing to perform the activity with the robot again and to provide their reasoning. To ensure session consistency and maintain a standardized timeline across all participants, they did not actually repeat the activity; however, their qualitative responses were recorded for qualitative analysis.

Our end-of-study survey measures included a demographics questionnaire for both the people living with dementia and their care partners, asking about age, gender, mindfulness habits, musical inclination, and prior experience with robots. The survey concluded with role-specific questionnaires. The demographic survey for the care partner included two additional questions gauging their recent emotional state. Care partners were asked to respond to the prompt ‘During the past week or so, I have,’ followed by two items: *Felt completely overwhelmed* and *Felt useful and needed*. The survey for people living with dementia concluded with the ADL/IADL questionnaires (Table 1). To respect their autonomy, people living with dementia were asked to fill out the ADL/IADL first, after which we verified their responses with their care partners.

**TABLE 1:** Activities of daily living (ADL) and instrumental activities of daily living (IADL) checklist.

ADL checklist	IADL checklist
Bathing	Using the telephone
Dressing	Preparing meals
Transferring (e.g., from bed to chair)	Managing household finances
Toileting	Taking medications
Grooming	Doing laundry
Feeding oneself	Doing housework
	Shopping
	Managing transportation

Subsequently, we analyzed the audio and video recordings of the session *post hoc* to characterize the nonverbal interaction dynamics, such as touching, talking, and waving. We focused on observing participant behaviors immediately after a robot action, under the assumption that the robot may have contributed to these behaviors. We noted and distinguished between participant interactions with the robot and those involving connecting with each other.

### 3.7 Analysis protocol

All qualitative analysis was conducted by a team of four researchers from the study team (henceforth C1, C2, C3, and C4). It is important to note the qualitative coders’ prior involvement in the data collection: C1 facilitated both Design Sessions and the User study, and C2 assisted with note-taking and facilitation in the second session. C3 had visited the care facility with C1 for volunteering outside of the research time, while C4 had never visited the facility. To ensure methodological rigor, specific analysis tasks were assigned to subsets of this team. The semi-structured interview transcripts were coded by C1 and C2, while C1, C3, and C4 performed the behavioral coding of recorded video data.

It is also important to note that responses were not obtained from every participant for every question in the study, as some participants with dementia had trouble understanding the researcher’s questions, or their CPs were occupied with providing immediate support. This is a known challenge with research with PLWD (Gierzynski et al., 2025) and must be taken into consideration when interpreting the results of this study.

#### 3.7.1 Semi-structured interviews

We manually transcribed all audio responses to the interaction questions listed in Table 2, as off-the-shelf systems are generally not trained to handle the speech patterns of PLWDs (Akinrintoyo et al., 2025). Following transcription, we analyzed the data and established several categories to encode based on recurring themes. The key categories coded were:

- *Difficulty with Robot Instructions*: Easy, Moderate, or Difficult, corresponding to questions *DB1*/*S1*
- *Participants’ Feeling During Activity*: Good, Fine, or Some Issues, based on questions *DB2*/*S2*
- *Robot Induced Discomfort*: Yes or No, based on questions *DB3*/*S3*
- *Willingness to repeat activity*: Yes or No, based on questions *DB4*/*S4*
- *Potential Place for Robot at Home*: Living Room, Bathroom, Dining Room, or Bedroom, in answer to question *E1*
- *Potential Use Frequency*: Everyday, A Few Times a Week, or Once a Week or Less, corresponding to question *E2*
- *Robot Induced closeness*: Yes, No (but reminder of close times), or No (no change), corresponding to question *E3*

**TABLE 2:** Semi-structured interview questions for deep breathing/singing activity and end of session.

Deep breathing/Singing activity		End of session	
Question ID	Question	Question ID	Question
DB1/S1	How easy or difficult was it to understand the robot’s instructions and follow along with the activity?	E1	If you had this robot at home, which room would you keep it in?
DB2/S2	How did you feel during the interaction with the robot?	E2	How often do you see yourself using the robot together with your care partner?
DB3/S3	Were there any moments when the robot made you feel uneasy or uncomfortable? If so, please describe them	E3	Did the robot make you feel closer to the other person?
DB4/S4	Would you be willing to engage in the activity with the robot again? Why or why not?	E4	What modifications would make the experience more useful or fun for you two?

To evaluate the consistency of the qualitative analysis, we calculated inter-coder reliability. Across all 12 items (4 questions across 3 interview phases), researchers’ agreement on which category to apply ranged from moderate to almost perfect (min: 0.452, max: 1.0). The mean Kappa score across all calculated items (excluding items with perfect, single-category agreement) was κ=0.719 , corresponding to substantial agreement. Detailed Kappa statistics per item are available in the Supplementary Material. Coders’ discrepancies in open-ended categorization were resolved through discussion. For ordered-scale categories, disagreements were resolved by retaining the more critical score to ensure a conservative lower-bound estimate. We also recorded their general feedback on the system, as *E4* served as an open-ended feedback prompt regarding modifications and was therefore not coded into specific themes.

#### 3.7.2 Post-hoc video analysis

To qualitatively characterize the interaction dynamics that emerged during the sessions, three researchers (C1, C3, and C4) annotated a subset of the video data. To ensure rigor, 6 of the 17 videos were coded by multiple researchers to establish inter-rater reliability (one by C1–C3–C4; two by C1–C3; two by C1–C4; one by C3–C4), while the remaining 11 videos were coded independently by a single researcher (C1: 3; C3: 4; C4: 4). For videos with multiple coders, disagreements in annotations were resolved through discussions among the coders; for the remaining videos, the single coder’s annotations were adopted as the final annotations. Coders used time-synced footage from both front- and side-facing cameras, selecting the angle that best showcased the behavior in question.

The coding protocol focused on capturing the nature of participants’ engagement in the context of the robot’s activity. We distinguished between two primary categories: (1) *Robot-Directed Behaviors*, where a participant’s attention or action was oriented toward the robot, and (2) *Interpersonal Behaviors*, where a participant’s engagement was directed toward their care partner or the person living with dementia. Across these two categories, we encoded behaviors that fell generally into one of the following types:

- *Affective and Emotional Expressions:* ranging from positive valence (smiling, laughing, giggling, and sighs of relief) to indicators of surprise, confusion, frowns, and occasional frustration.
- *Vocal and Melodic Engagement:* encompassing full singing, soft humming, lip-syncing (mouthing lyrics), harmonizing with the robot or partner, and rhythmic vocalizations.
- *Non-Verbal Social Cues:* including mutual glancing, sustained eye contact, nodding in agreement, and shifting gaze between the robot, the partner, and the tablet interface.
- *Tactile Interaction and Anthropomorphism with the Robot:* manifested through petting, squeezing, or rubbing the robot’s sweater, as well as verbalizing greetings and using gendered pronouns to refer to the device.
- *Physical and Rhythmic Synchrony:* such as swaying to the music, tapping feet or hands to the tempo, nodding in rhythm, and subconsciously mirroring the partner’s posture or movements.
- *Affection and Proximity:* characterized by handholding, embracing, leaning toward the partner, and kissing, reflecting the level of intimacy and comfort within the dyad.
- *Care Partner Scaffolding:* involving the care partner providing verbal instructions, physical redirections, nudging to encourage participation, and assisting the person living with dementia with task-related movements.
- *Cognitive and Attentional Behaviors:* including spontaneous memory recall triggered by the activity, focused observation of the robot’s movements, and seeking clarification on instructions.

We encoded the lowest level information (e.g., smiling, hand holding, memory recall) with timestamps for each video. This information was paired with who performed the behavior (PLWD or CP), whether the behavior was targeting the robot or the other human, and what the most recent robot action was. Inter-rater reliability was assessed using Cohen’s Kappa for videos with two coders and Fleiss’ Kappa for videos with three coders. These metrics were calculated for each coding dimension (observed behavior target, robot action, human actor), and a combined measure across all dimensions. All reliability measures met or exceeded the 0.40 threshold for acceptable inter-rater reliability in content analysis (Landis and Koch, 1977). A detailed κ breakdown can be found in the Supplementary Material. Finally, to interpret the aggregated observational data, we performed a thematic analysis on the qualitative coding logs to identify recurring themes. These are discussed in Section 4.2.

## 4 Results

This section presents results from post-activity and end-of-session interviews, followed by a *post hoc* video analysis organized into four themes: Intimacy and Synchrony, Humor and Playfulness, Recall of Past Events, and Negotiating Preferences.

### 4.1 Semi-structured interviews

For each study session, we conducted semi-structured interviews after each activity (deep breathing and singing) to gather participant feedback on those specific interactions. A final interview was then held at the end of the session to capture overall feedback on the system.

#### 4.1.1 Pos-activity interviews

We organized the qualitative responses from the semi-structured interviews to describe participants’ experiences with the deep breathing (DB) and singing (S) activities. Detailed visualization of interview responses is provided in the Supplementary Material. Note that the total number of responses reported varies slightly across categories due to occasional non-responses; all counts and percentages are calculated based on the number of responses actually provided.

##### 4.1.1.1 DB1 and S1: ease of understanding and following instructions

Participants generally found the robot’s instructions easy to follow across both activities. For Deep Breathing, the majority of care partners (15/17) and people living with dementia (10/17) described the activity as straightforward. This was particularly true for the four participants with dementia who had prior experience with yoga or chanting, as they found the robot-led session familiar. For Singing, accessibility was similarly high, with a substantial majority of pairs (15/17 PLWD; 13/17 CP) rating it as “easy” or “very easy.” Care partners frequently noted that the visual lyric cues on the tablet were instrumental in this success.

Despite the general ease, specific sensory challenges emerged for each activity. During breathing, several pairs noted that the auditory chimes were too soft. During singing, the primary critique (noted by 4/17 PLWD and 2/17 CPs) concerned the robot’s audio quality; participants described the voice as “excessively robotic,” which was particularly challenging for one care partner (P16) with hearing aids.

##### 4.1.1.2 DB2 and S2: feelings during activities

When asked how they felt during the sessions, participants differentiated between the “calm” of breathing and the “fun” of singing.

- Deep Breathing: Most pairs (13/17 PLWD; 15/17 CPs) reported feeling “fine” or “good,” with five pairs specifically describing it as “relaxing.” Three participants with dementia highlighted that the tactile sensation of the robot’s expanding body provided a helpful calming cue. However, the calming effect for P7’s care partner was diminished by interpersonal distractions, specifically when the PLWD placed an arm around the CP during the robot’s movement. Additionally, pair P6 noted that the ‘hold’ phase was too long, making it difficult to maintain the pace due to their specific health constraints.
- Singing: The emotional response to singing was characterized by enjoyment and connection. The majority of participants (9/17 PLWD; 10/17 CPs) reported feeling “good”, three of whom described the experience as “fun,” “cute,” or “joyful.” Care partners (e.g., P2, P15) emphasized the happiness they felt witnessing their partner’s engagement, and several viewed the activity as a meaningful opportunity to reconnect through a shared hobby they rarely practice anymore. A smaller group (6/17 PLWD; 3/17 CPs) reported feeling “fine,” and (2/17 PLWD; 2/17 CP) reported experiencing “some issues.” Notably, both participants from P17 expressed dissatisfaction, noting that they simply do not like to sing.

##### 4.1.1.3 DB3 and S3: perceived comfort and unease from the robot

We had very few reports of people feeling uncomfortable around the robot. The vast majority of participants reported no discomfort during breathing (16/17 PLWD, 16/17 CP) and singing (15/17 PLWD, 16/17 CP). When “unease” was reported, it was generally related to confusion or performance anxiety rather than fear of the robot.

- Deep Breathing: A few participants living with dementia expressed minor confusion about the flow of the activities, while some care partners felt initial skepticism that dissipated as the session progressed. A person living with dementia from P11 specifically praised the robot’s non-verbal cues, noting they liked “the way it goes up and down and the eyes closing.”
- Singing: Discomfort was largely linked to pacing. One pair noted the lyrics moved too fast, and P1 found the song *“Singing in the Rain”* physically challenging to keep up with, as it was too high-pitched. Although one care partner (P2) typically found robots uncanny, they found this specific robot’s blinking to be amusing, while another care partner (P10) became emotional, though not uncomfortable.

##### 4.1.1.4 DB4 and S4: willingness to repeat sessions

People were excited about using this robot in the future. All 17 care partners expressed a willingness to repeat the Deep Breathing activity, valuing the potential for mindfulness and routine. Similarly, 15 of 16 people living with dementia were willing to continue, finding the session calming. Person living with dementia from P3 initially declined but changed their response to “yes” upon being prompted for further elaboration, though they appeared somewhat confused by the question. Notably, social motivation influenced participation; in pair P9, the care partner agreed to repeat the activity solely for her mother’s benefit, despite it not being her personal preference.

For Singing, willingness to repeat the activity remained high, though slightly more varied. Most pairs (14/17 PLWD, 14/16 CP) responded with a clear “yes,” with care partners viewing it as a low-pressure, enjoyable activity for connection. However, preferences played a larger role here: the person living with dementia in P3 initially found it repetitive before reconsidering, while both participants in P17 declined the future possibility of singing together with the robot entirely, citing a long-standing mutual dislike of singing.

#### 4.1.2 End of session interviews

Following the activities, we conducted semi-structured interviews to gauge participants’ interest in home deployment and gather feedback on potential integration into their daily lives. The exact wording of the questions can be found in Table 2. Detailed visualization of interview responses is provided in the Supplementary Material.

##### 4.1.2.1 E1: robot placement at home

When asked where they would keep the robot, the majority of pairs favored central, shared spaces such as the living room or family room (10/13 PLWD; 10/15 CPs), noting these are natural hubs for relaxation. A smaller subset (1/13 PLWD; 3/15 CPs) suggested the dining room, often citing its proximity to other daily routines.

However, privacy and household logistics influenced other preferences. A few participants considered more private areas of the house, such as the bedroom (1/13 PLWD; 2/15 CPs) or the bathroom (1/13 PLWD). For example, P12’s care partner suggested a spare bedroom to reduce distractions, specifically highlighting the need to keep the robot “somewhere out of the dog’s way.” For pairs living apart (e.g., in assisted living), communal areas were identified as the most practical location for shared use.

##### 4.1.2.2 E2: robot usage frequency at home

Projections for usage frequency varied significantly. Many participants (10/15 PLWD; 7/16 CPs) reported seeing themselves using the system daily, viewing the robot as part of a regular health routine. One person living with dementia stated they would like to *“try it every day,”* while a care partner echoed this, suggesting use up to *“twice a day”* like an exercise regimen.

Conversely, others viewed it as an occasional activity. Some participants preferred a weekly cadence, such as wanting to *“sing on Saturday nights,”* or treating the robot as a “guest” to show friends (P7). Preferences also varied by activity type; P7 noted they would perform deep breathing more often than singing, suggesting it would be nice to vary the number of breaths they could do. Finally, practical barriers, such as not cohabitating, led some care partners (3/16) to predict limited personal usage.

##### 4.1.2.3 E3: closeness to the other person

All pairs of people living with dementia and their care partners entered the study with strong relationships developed over decades. When asked if the robot made them feel closer, many pairs (9/16) affirmed that the shared experience, particularly singing, enhanced their connection. Two pairs indicated that the session served to validate an existing bond rather than create a new one. For example, the care partner from P4 noted, *“(We are) already close to begin with, but feel more connected because it was fun and we can be goofy about it.”* Similarly, the care partner from P10 remarked that the activity “*reminds me of how close we are when we sing together but didn’t (make me) directly feel closer.”* Five of the pairs felt no perceived change in their level of closeness. P1 and P11 noted that they were already very close to begin with.

##### 4.1.2.4 E4: proposed improvements to enhance the robot experience

When asked, “*What will make the experience more useful or fun for you two?*”, participants suggested several specific improvements:

1. *Interactive Visualizations:* Two participants suggested ways to improve the visual interactivity of the system. The care partner from pair P5 suggested “*more things to pop up on the screen when singing*” and the inclusion of stories with accompanying visualizations, along with the option to “*pause and resume whenever.*” Another care partner (P16) proposed integrating video directly into the robot because it was “*difficult for my partner to concentrate on two things at once.*”
2. *Improved Tactile and Auditory Feedback:* Participants also expressed interest in more sensory features. The care partner from pair P12 suggested adding “*calming background music during deep breathing*,” and the care partner from pair P5 suggested adding “*more tactile feedback, vibrations, and responsive feedback when you touch the robot.*”
3. *Need for Personalization:* Some participants (4/17) suggested more personalized and familiar music. One person living with dementia (P4) specifically requested “*more songs that she knows*,” while the care partner from pair P8 expressed interest in “*more songs, like Beatles songs.*” One care partner from pair P7 also suggested that they would prefer to tailor the number and duration of the breaths for the deep breathing interaction.
4. *Reminders and Engagement:* Two care partners proposed expanding the robot’s practical utility to better support daily living. The care partner from P17 suggested that personalized reminders would significantly increase the robot’s value, stating that if the device could provide auditory and visual cues—such as, “*Today is Monday, it’s garbage day!”* or reminders regarding phone navigation—it would be substantially more useful. Similarly, the care partner from P5 suggested a shared leisure feature, noting that if the robot could read books aloud, it would allow the couple to enjoy a stationary activity together.

### 4.2 Post-hoc video analysis

**FIGURE 7:**
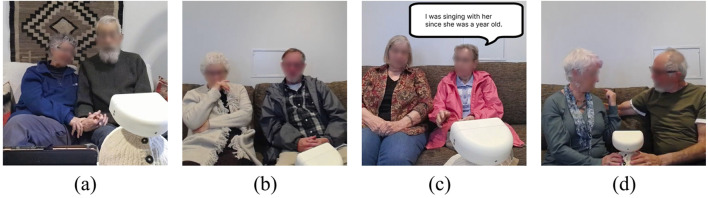
Examples of interactional themes. **(a)** Intimacy and Synchrony: participants holding hands while singing; **(b)** Humor and Playfulness as Coping: a person living with dementia covering her mouth after singing too early; **(c)** Recall of Past Events: a person living with dementia recounting a history of singing with their care partner; and **(d)** Negotiating Preferences: a care partner attempting to redirect a person living with dementia’s hands from her shoulder to the robot during a deep breathing activity.

To capture the nuances of shared engagement, three researchers (C1, C3, C4) annotated a total of 1,515 interaction events ( M=89.1 per session, SD=40.6 ), defined as observable social behaviors (e.g., gazing, talking, touching, holding hands, or waving) directed toward the robot or a partner. Subsequently, we filtered the data to retain only those interactions that occurred while participants were actively doing deep breathing or singing activities. This process resulted in a final dataset of 894 interaction events ( M=52.6 per session, SD=35.2 ), which were categorized according to the protocol in Section 3.7.2. Based on a thematic analysis of these events, we identified four notable themes: (a) Intimacy and Synchrony, (b) Humor and Playfulness as Coping, (c) Recall of Past Events, and (d) Negotiating Preferences, which are consistent with established social dynamics observed in human-led music therapy and shared deep breathing interventions for people living with dementia and care partners (Berk et al., 2019; Stedje et al., 2023). Figure 7 shows a visual example of each theme.

Given the exploratory nature of this work, these categories are not intended to represent a universal experience, but rather to illustrate possibilities of dynamics that emerged during the interaction. Accordingly, we highlight these themes to characterize the breadth of social moments that are possible in this setting, suggesting that while the robot provides a shared context. We recognize that in real interactions, these themes often overlap; for example, a joke can be a way of showing closeness. To address this, we defined each theme by its main purpose: whether the moment was primarily about emotional connection, lightening the mood when “mistakes” occurred, sharing a past memory, or navigating moments where partners had different preferences.

#### 4.2.1 Intimacy and synchrony

Each person living with dementia and their care partner entered the study with a strong relationship that had developed over many years. During both the singing and breathing activities, we observed 8/17 pairs of people living with dementia (PLWD) and their care partners (CP) affectionately touching each other and/or synchronizing movements. Explicit expressions of affection often occurred during singing activity; for example, in pair P3, the PLWD kisses the CP, and in P5, the PLWD caresses CP’s arm and face.

Beyond these explicit gestures, intimacy also manifested as subtle, comforting micro-interactions. Some pairs maintained consistent physical contact, seemingly for reassurance or guidance. For example, the pair in P12 sat with their thighs and shoulders touching throughout the session. In P14, the CP affectionately traced their finger around the PLWD’s hand while holding it, and in P15, a playful interaction led the pair to interlace their fingers. Similarly, in P12 and P15, participants grabbed their partner’s hands immediately upon finishing each activity.

During singing, some pairs frequently gesture in sync to the rhythm of the song: P13 swayed their heads and bodies in sync, eventually moving physically closer, and P12 linked arms to sway side-to-side. This connection also extended to the deep breathing phase. In P14, the specific act of holding the robot together created a moment of connection, during which both glanced at each other and smiled. Similarly, during deep breathing, people living with dementia from P5 and P2 frequently looked at their partners while trying to match their breathing rhythm. This suggests they were not just following the robot but were relying on each other’s gaze for cues and comfort during the exercise.

#### 4.2.2 Humor and Playfulness as coping

We observed 5/17 pairs of participants laughing and joking in the face of mistakes, which appeared to transform potential embarrassment into playful moments of joy. “Performance errors” frequently triggered shared amusement rather than frustration. For example, in pair P1, the PLWD laughs at the CP for singing at the wrong time, then covers her mouth and laughs shyly when she realizes she is singing ahead of the robot. Similarly, pair P3 laughed at each other for singing off-key, while pair P12 giggled when they realized that they were singing ahead of the beat. In P17, when the PLWD self-deprecated that he “wishes he could sing better,” the CP laughed along with him, validating the effort over quality.

Sometimes, participants engaged in playful rebellion or physical humor to assert competence. The PLWD in pair P9, jokingly, gestures as if she is going to touch the robot again after being told not to, playfully pushing boundaries. Care partners also used physical levity to maintain engagement: in P4, the CP jokingly nudged the PLWD’s shoulder in time with the music, and in P13, the CP laughed at her own exaggerated hand gestures. Beyond having fun, these moments, such as in P14 when laughter broke the silence during deep breathing, seemed to ease tension by dropping their shoulders. These activities appeared to allow the pairs to approach the interaction more lightly, focusing on their shared connection rather than strictly following the task.

#### 4.2.3 Recall of past events

After reviewing the transcript of the entire interaction, we observed that some people living with dementia (4/17 sessions) fondly recalled memories during the singing interaction. In Pair P6, where both partners are living with dementia, the wife (care partner) prompted her husband’s memory, asking: “*Remember we used to sing that all the time from Sesame Street with the kids? Letter B … Letter B,*” to which her husband enthusiastically responded, “*Yes, of course yes!”* This theme of remembering family activity was echoed by P5, who remarked, “*We used to have little kids coming over and doing music together all the time.”* For others, the activity highlighted a renewed connection. The person living with dementia in P11 shared, “*[This is the] first time I sang out loud with my wife in quite a while … I feel good,”* noting that he did not “*remember the last time we sang together.”* Similarly, the mother in P9 (PLWD), who lives apart from her daughter, emphasized their enduring bond through music: “*We don’t see each other very often, but when we do see each other, we sing. I was singing with her since she was a year old.”*

#### 4.2.4 Negotiating preferences

We observed a little friction between the care partner’s desire for variety and the person living with dementia’s preference for familiarity. This was particularly evident during song selection, where 4/17 pairs repeated the same song more than once despite the CP’s attempts, such as singing snippets (P14), guiding hands (P16), or verbally prompting (P13), to encourage new choices. However, this push for novelty often gave way to the benefits of familiarity and repetition. In P3, the PLWD selected the same song twice despite the CP’s initial hesitation. Similarly, in P2, after the PLWD withdrew during a new song, the CP pivoted back to their first choice. This decision immediately restored the PLWD’s participation, allowing them to sing together for the rest of the track.

Beyond song selection, pairs sometimes had different preferences for how they should approach the activity. For example, during deep breathing, PLWD in P7 wrapped his arm around CP’s shoulder to connect. Still, the CP noted that this contact made it hard to focus on deep breathing, highlighting a trade-off between connection and task adherence for activities that are more introspective or more directed towards the robot. Similarly, in P16, the pair negotiated their mode of participation during the singing phase; while the CP encouraged the PLWD to sing along, the PLWD preferred to drum along to the beat rather than sing. Thus, the pair settled into a method of engagement with our system in which they enjoyed the music together through parallel yet distinct behaviors.

## 5 Discussion

In this exploratory study, our observations suggest that the robot’s programmed activities provided a structure for shared physical and social engagement, such as touch, humor, and conversation. However, it is important to note that this study was exploratory and lacked a non-robotic control condition; therefore, we cannot definitively conclude that these interactions are unique to their interaction with the robot. These moments of connection may stem from the novelty of the technology or the structured nature of the activities themselves. Within this context, we use these observed interactions to frame three opportunity spaces for future HRI research involving the robot-PLWD-CP triad.

### 5.1 The robot as a focus for synchronization

One core component of “togetherness” that we observed repeatedly was synchronicity (Section 4.2.1), where the robot provided a shared structure, such as a breathing rhythm or song, for the pair to follow. This pattern implies that the robot can function as a shared external anchor. While PLWDs still often required assistance, the robot’s guidance offered a structure that appeared to support CPs in momentarily stepping back from an instructional role to engage as a co-participant. We suggest future designs might prioritize multimodal cues, such as haptic pulses or visual breathing guides, that allow the pair to sync their focus intuitively, minimizing the need for verbal correction between humans. The critical trade-off, however, lies in the balance of attention. While increasing the robot’s social guidance could further support the PLWD, doing so risks drawing too much attention to the device itself. If the robot becomes too dominant a social actor, it may disrupt the shared attention between the two people, transforming a “together” moment into two separate interactions with a machine. By positioning the robot as a shared anchor rather than a replacement for human presence, our observations suggest how such technology might scaffold shared participation in mixed-ability pairs. This highlights a broader opportunity for robots to move beyond individual-focused support, instead exploring their potential to facilitate richer, continuous connections for people living with dementia and care partners.

### 5.2 The robot as a scaffold for joint play

Beyond the functional mechanics of the activities, we observed that the sessions frequently inspired moments of shared humor and “play” (Section 4.2.2). This serves as another form of togetherness between PLWDs and CPs with our designed interactions. While we did not necessarily design the robot to be playful itself (rather to act more as a guide), we observed that participants appeared comfortable making mistakes in its presence, often treating errors playfully rather than with embarrassment. The singing activity, in particular, allowed PLWDs and their CPs to reconnect through familiar songs in a fun and joyful way. We were heartened to observe participants pulling pranks with the robot or laughing at their own mistakes during this activity. Multiple participants also noted a desire for more play-oriented features like jokes and riddles. We suggest that future designs consider leaning where the robot can use its own agency to promote joint moments of humor and play based on the activity at hand. However, this requires a balance between play and respect. If the robot is too playful, it risks treating users like children or distracting them from the instructions. Future designs must ensure that the robot’s behavior supports humor between adults without becoming confusing or disrespectful. The playful interactions observed in this study show that the robot can be more than just a tool for managing symptoms or achieving clinical goals. Instead, these observations highlight a broader potential to design technology that supports the wellbeing of both people living with dementia and their care partners, prioritizing their emotional connection.

### 5.3 The robot as a source of both familiarity and variety

A frequent dynamic observed in the sessions was the negotiation between the comfort of familiarity and the desire for variety (Section 4.2.4). While care partners often encouraged people living with dementia to try a new song, they typically yielded to the PLWD’s preference for repetition, which restored the PLWD’s participation and confidence. However, this could provide tension for the CP if they desire novelty for themselves, or if they believe that it is beneficial for the PLWD to experience new things. Similarly, we observed tension between performing the deep breathing activity as instructed (e.g., placing hands on the robot while deep breathing) and the familiar comfort of physical touch (e.g., rubbing another person’s shoulder).

In these ways, the robot’s interactions could weaken the sense of togetherness, based on how it navigates the established tendency that PLWDs have for the familiar. When designing our system, we had intended to lean into the familiar to bring PLWDs and CPs together through known deep breathing patterns and songs. However, even within these activities, there were differences among PLWDs and their CPs for how to approach them. We suggest that future interaction designs consider how the robot can mediate these differences. Contextual awareness of what each part of the pair desires could help the robot know when to encourage the familiar versus variety. There is a delicate balance in this mediation: if the robot leans too far toward repetition, it might not be engaging for the CP and may fail to challenge the PLWD. Conversely, if the robot leans too far toward unfamiliar activities, it may become too difficult for the PLWD to follow along, potentially disrupting the interaction. This tension highlights the need to design for both people living with dementia and care partners, rather than centering solely on the person living with dementia. It points toward a broader direction for exploring how robots can balance providing comfort for one person while offering variety for the other, helping the pair navigate activities as a dynamic team.

## 6 Limitations and future work

While our interaction design sessions and user study yielded preliminary insights, several limitations emerged regarding the system’s readiness for in-home deployment. Currently, the robot lacks the robust environmental sensing required to handle uncontrolled domestic settings, where ambient noise may interfere with voice commands and variable lighting can reduce display visibility. To address these challenges, future development will focus on integrating non-contact sensors and smart-home connectivity to monitor user states and environmental context actively (Georgiou et al., 2023; Ghose et al., 2024). This will enable the robot to reason about contextual signals to automatically modify its behavior, such as adjusting volume, display contrast, and interaction timing, to suit the immediate situation. Additionally, the reliance on manual initiation places a setup burden on care partners. Participants also suggest implementing predictive scheduling that syncs with voice assistants to identify optimal engagement times, helping users engage with the robot more often. Future iterations should also consider incorporating new functionalities that promote shared interaction, enabling the robot to serve as a daily assistant for medication and chore reminders that the care partner and person living with dementia can manage together. As this study was limited to a single session, it may not capture evolving relationship dynamics. Future work should involve longitudinal deployments, which are proven to be effective for understanding long-term system adoption and usage patterns (Matheus et al., 2025a; Matheus et al., 2026; Hu and Šabanović, 2026). Finally, our study included two pairs where both individuals were living with dementia, yet one continued to act as a care partner for the other. As this population is understudied, future work should explore their interactions with robots to identify design opportunities that support their shared agency.

## Data Availability

The datasets presented in this article are not readily available because the study protocol (approved by the institutional review board) and verbal consent from participants to be in the study does not include a statement relating to third party access to participants’ data. Requests to access the datasets should be directed to Jirachaya Fern Limprayoon, fern.limprayoon@yale.edu.
