# Supplementary material for: Exploring robot-led activities between people living with dementia and family care partners
Source: Front Robot AI. 2026 Apr 28;13:1772079. doi: 10.3389/frobt.2026.1772079 (PMC13172625; doi:10.3389/frobt.2026.1772079)
Supplement: Supplementary file 1 [file DataSheet1.pdf]

Supplementary Material

1 SEMI-STRUCTURED INTERVIEW

This section provides the detailed breakdown of the semi-structured interview responses collected during the user study. For each study session, interviews were conducted immediately following each activity and at the conclusion of the session using the questions listed in Table S1 to capture immediate impressions and potential for in-home use.

Table S1. Semi-Structured Interview Questions for Deep Breathing/Singing Activity and End of Session

**Deep Breathing / Singing Activity**		**End of Session**	
**Question ID**	**Question**	**Question ID**	**Question**
**DB1 / S1**	How easy or difficult was it to understand the robot's instructions and follow along with the activity?	**E1**	If you had this robot at home, which room would you keep it in?
**DB2 / S2**	How did you feel during the interaction with the robot?	**E2**	How often do you see yourself using the robot together with your care partner?
**DB3 / S3**	Were there any moments when the robot made you feel uneasy or uncomfortable? If so, please describe them.	**E3**	Did the robot make you feel closer to the other person?
**DB4 / S4**	Would you be willing to engage in the activity with the robot again? Why or why not?	**E4**	What modifications would make the experience more useful or fun for you two?

Below is the table of inter-coder reliability per coded items for each semi-structured interview question.

Table S2. Inter-Coder Reliability (Cohen's Kappa) for Semi-Structured Interview Question Categories

Question Category	Item ID	Kappa Type	CP Rating		PLWD Rating	
			Cohen's κ	N	Cohen's κ	N
**Deep Breathing (DB) / Singing (S)**						
**Difficulty**	DB1	Weighted (Q)	0.646	17	0.573	17
**Understanding Instruction**	S1	Weighted (Q)	0.841	17	0.935	17
**Feeling during Activity**	DB2	Weighted (Q)	0.589	17	0.810	17
	S2	Weighted (Q)	0.585	17	0.679	17
**Willingness to Repeat**	DB4	Nominal	N/A*	17	1.000	16
	S4	Nominal	0.636	16	0.452	17
**End of Session (E)**						
**Room Placement**	E1	Nominal	0.886	15	1.000	13
**Expected Use Frequency**	E2	Weighted (Q)	0.760	16	0.789	15
**Closeness**	E3 (Combined)	Weighted (Q)	0.763 ($N = 17$)			

CP: care partner, PLWD: person living with dementia. Interview Questions are defined in Table S1. Q: Quadratic weighting applied. N/A*: Cohen's Kappa could not be calculated due to 100% agreement on a single category, thus indicating Perfect Agreement ($\kappa = 1$).
This applies to CP:DB4 and all *Discomfort with Robot* items (CP:DB3, PLWD:DB3, CP:S3, PLWD:S3).

The two following figures organize the data to allow for a direct comparison between Care Partners (CP) and People Living with Dementia (PLWD) in 17 pairs of participants. Capturing both voices is essential as care partners and people living with dementia often have different perspectives on preferences and perceptions towards the system.

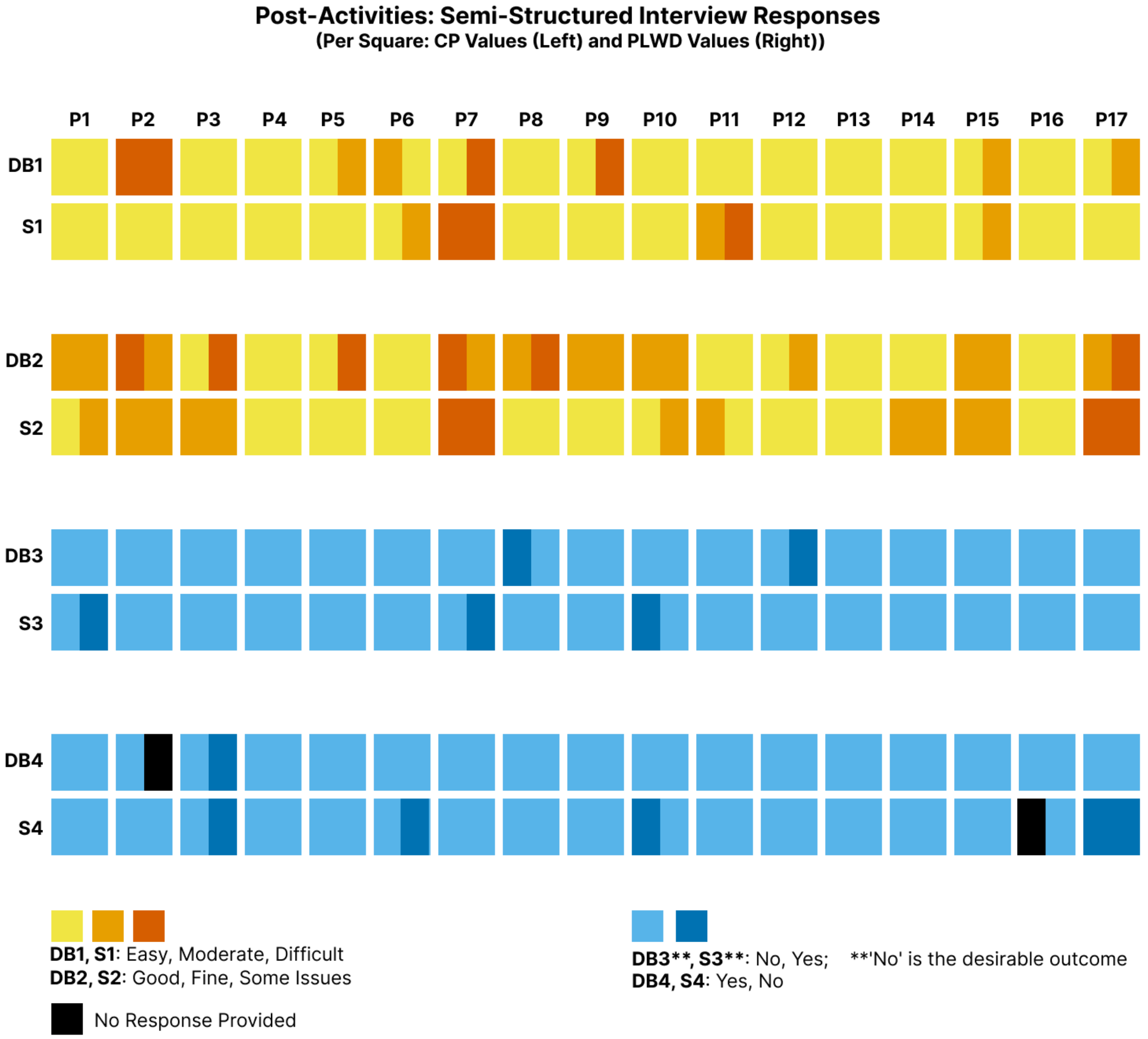

Figure S1: **Post Activities: Qualitative Coding Results for Semi-Structured Interview Responses.** This figure presents responses by participant pair ID (column) and Question ID (row). Each cell contains the care partner's (CP) response (left) and the person living with dementia's (PLWD) response (right). See Table S1 for complete question details.

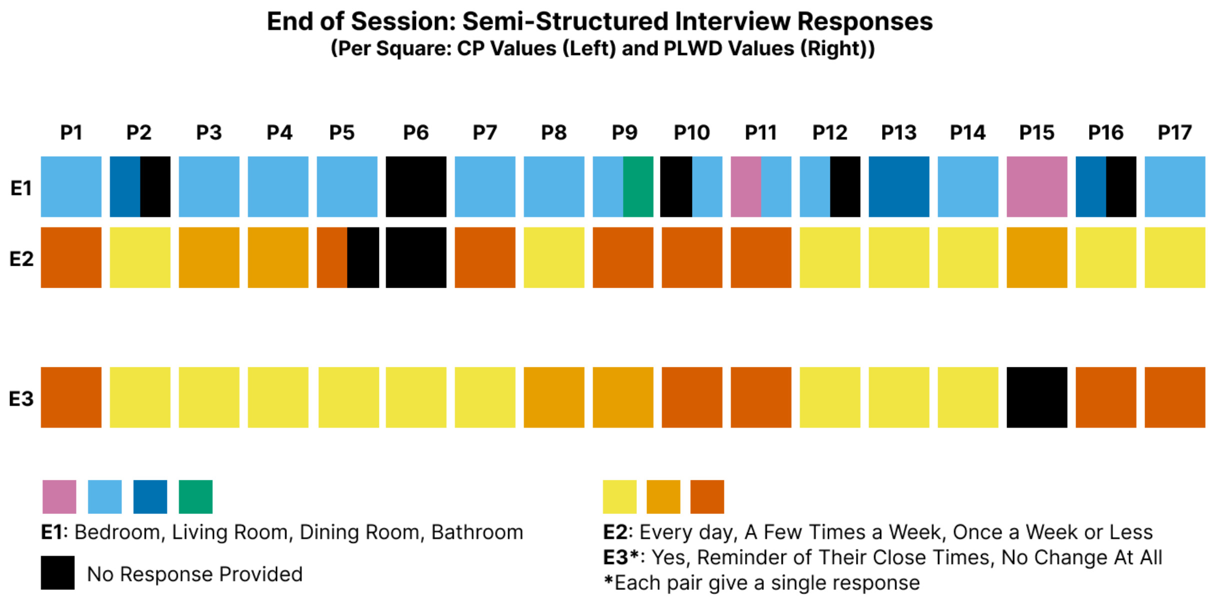

Figure S2: **End of Session: Qualitative Coding Results for Semi-Structured Interview Responses.** This figure presents responses by participant pair ID (column) and Question ID (row). Each cell contains the care partner's (CP) response (left) and the person living with dementia's (PLWD) response (right). See Table S1 for complete question details.

2 POST-HOC VIDEO ANALYSIS

To qualitatively assess interaction dynamics, three researchers (C1, C3, C4) annotated 17 videos (24 annotations each), excluding the introspective deep breathing phase. To ensure rigor, 6 videos were cross-coded by multiple researchers to establish inter-rater reliability, while the remaining 11 were coded independently (C1: 3; C3: 4; C4: 4). Below is a table detailed inter-coder reliability breakdown.

Table S3. Inter-rater Reliability Statistics by Coder Group. Coders were grouped based on the participant dataset they evaluated.

Coder ID	Interaction Type (κ)	Robot (κ)	Human Actor (κ)	Combined (κ)	Human Reaction Agreement (%)
C1-C3	0.681	0.696	0.703	0.568	67.85
C1-C4	0.689	0.541	0.719	0.518	67.50
C3-C4	0.566	0.409	0.636	0.442	59.00
C1-C3-C4	0.599	0.555	0.633	0.519	65.90
**Mean**	**0.634**	**0.550**	**0.673**	**0.512**	**65.06**
